# *ClPH4-like* coordinately regulates citric acid and anthocyanin accumulation in purple wampee

**DOI:** 10.1093/hr/uhag182

**Published:** 2026-05-04

**Authors:** Zhihao Lu, Li Liao, Juan Sun, Bin Hu, Jialing Fu, Gang Hu, Tao Yuan, Kun Yang, Yuping Huang, Wang Zhang, Yuantao Xu, Xia Wang, Fangfang Wu, Xiuxin Deng, Qiang Xu

**Affiliations:** National Key Laboratory for Germplasm Innovation & Utilization of Horticultural Crops, College of Horticulture and Forestry Sciences, Huazhong Agricultural University, Wuhan 430070, China; National Key Laboratory for Germplasm Innovation & Utilization of Horticultural Crops, College of Horticulture and Forestry Sciences, Huazhong Agricultural University, Wuhan 430070, China; National Key Laboratory for Germplasm Innovation & Utilization of Horticultural Crops, College of Horticulture and Forestry Sciences, Huazhong Agricultural University, Wuhan 430070, China; National Key Laboratory for Germplasm Innovation & Utilization of Horticultural Crops, College of Horticulture and Forestry Sciences, Huazhong Agricultural University, Wuhan 430070, China; College of Agriculture, Guangxi University, Nanning 530004, China; National Key Laboratory for Germplasm Innovation & Utilization of Horticultural Crops, College of Horticulture and Forestry Sciences, Huazhong Agricultural University, Wuhan 430070, China; National Key Laboratory for Germplasm Innovation & Utilization of Horticultural Crops, College of Horticulture and Forestry Sciences, Huazhong Agricultural University, Wuhan 430070, China; National Key Laboratory for Germplasm Innovation & Utilization of Horticultural Crops, College of Horticulture and Forestry Sciences, Huazhong Agricultural University, Wuhan 430070, China; National Key Laboratory for Germplasm Innovation & Utilization of Horticultural Crops, College of Horticulture and Forestry Sciences, Huazhong Agricultural University, Wuhan 430070, China; National Key Laboratory for Germplasm Innovation & Utilization of Horticultural Crops, College of Horticulture and Forestry Sciences, Huazhong Agricultural University, Wuhan 430070, China; National Key Laboratory for Germplasm Innovation & Utilization of Horticultural Crops, College of Horticulture and Forestry Sciences, Huazhong Agricultural University, Wuhan 430070, China; Hubei Hongshan Laboratory, Huazhong Agricultural University, Wuhan 430070, China; National Key Laboratory for Germplasm Innovation & Utilization of Horticultural Crops, College of Horticulture and Forestry Sciences, Huazhong Agricultural University, Wuhan 430070, China; Hubei Hongshan Laboratory, Huazhong Agricultural University, Wuhan 430070, China; Science and Technology Innovation Research Center of Majia Pummelo, Guangfeng, Shangrao, 334000, China; National Key Laboratory for Germplasm Innovation & Utilization of Horticultural Crops, College of Horticulture and Forestry Sciences, Huazhong Agricultural University, Wuhan 430070, China; Hubei Hongshan Laboratory, Huazhong Agricultural University, Wuhan 430070, China; National Key Laboratory for Germplasm Innovation & Utilization of Horticultural Crops, College of Horticulture and Forestry Sciences, Huazhong Agricultural University, Wuhan 430070, China; Hubei Hongshan Laboratory, Huazhong Agricultural University, Wuhan 430070, China

## Abstract

Citric acid and anthocyanins are key metabolites that determine fruit flavor and coloration. Although the accumulation mechanisms of citric acid and anthocyanins are known in citrus, little is known about the cross-regulatory mechanism between the two important metabolites. In this study, we identified a purple wampee (*Clausena lansium*) that exhibits simultaneous accumulation of citric acid and anthocyanins in fruit. Functional analyses confirmed that the *ClPH4* positively regulate citric acid accumulation; however, the homologous gene of *Ruby1*, the activator of anthocyanin biosynthesis, has lost the capacity to induce anthocyanin accumulation in the purple wampee. We subsequently identified a *PH4-Lik*e based on gene family and expression analyses. Stable overexpression of *ClPH4-Like* in citrus, along with transient silencing in purple wampee pulp, demonstrated that it can simultaneously induce both anthocyanin and citric acid accumulation. Moreover, *PH4-Like* is specifically and highly expressed in purple wampee, consistent with the observation that its promoter activity is much higher in purple wampee than in other citrus species. Biochemical assays showed that PH4-Like binds to the promoters of *Flavonoid 3′-Hydroxylase* (*F3′H*), *Dihydroflavonol 4-reductase* (*DFR*), *Anthocyanidin synthase* (*ANS*) and the proton pump genes *PH5* and *VHA-a2*, the key genes in the anthocyanin and citric acid accumulation. Together, our findings uncover a key gene that coordinately regulates citric acid and anthocyanin accumulation, providing insight into the genetic events involved in the diversification of fruit color and taste in Aurantioideae, and offering valuable targets for simultaneous improvement of fruit quality.

## Introduction

Citric acid is the predominant organic acid in citrus fruits [1] and is primarily responsible for their sour taste. An appropriate content of citric acid can maintain fruit flavor and prevents it from becoming bland. Anthocyanins are major contributors to fruit coloration [2] and possess a range of biological activities, including antioxidation and attenuation of aging and obesity [3]. Several horticultural crops exhibit a pattern of coordinated accumulation of high levels of organic acids and anthocyanins, but the underlying mechanisms linking these traits remain unclear [4, 5]. In the *Citrus* genus, the coordinated accumulation of acid and pigmentation exhibits tissue-specific coupling [6], but citrus fruits pulp simultaneously accumulating both citric acid and anthocyanins have not been reported.

Key regulatory factors controlling organic acids in major horticultural crops have been elucidated [7]. In apple, *MdMYB73* has been shown to positively regulate malic acid accumulation by upregulating the expression of the malate transporter *MdALMT9* [8] and the tonoplast proton pump genes *MdVHA-A* and *MdVHP* [9]. Moreover, advances have also been achieved in elucidating the upstream environmental signaling pathways that regulate *MdMYB73* [10]. In tomato, organic acids are predominantly composed of malic acid and citric acid [11], with *ALMT9*, an organic acid transporter, also playing a crucial role [12]. PH4 has been identified as a major transcription factor controlling citric acid accumulation in citrus [13]. The knockout of *PH4* results in a complete loss of citric acid accumulation in citrus fruit. Its promoter variations may explain the emergence of citric acid accumulation during the diversification of the Aurantioideae. In addition, PH4 not only activates *PH5* expression but also induces *TRL*, which modulates citric acid accumulation by repressing transactivation activity of *PH4* to prevent excessive accumulation of citric acid [14]. Besides, the transcriptional regulation of anthocyanin biosynthesis has been extensively studied. Since the discovery in the 1960s that *C1*(MYB) in maize positively regulates anthocyanin accumulation [15, 16], key regulators identified in various other plant species have largely been homologs of *C1*, such as *Arabidopsis PAP1* [17], citrus *Ruby1* [18], apple *MYB1* [19], and strawberry *MYB10* [20]. This indicates that the function of this S6 subgroup in the MYB family promoting anthocyanin accumulation is highly conserved in plants. In addition, preliminary studies have explored the relationships among different coloration patterns and, separately, among distinct flavor profiles. For example, in citrus *BBX22* has been shown to positively regulate the accumulation of both carotenoids and anthocyanins [21], while the citrus *SAR* gene can simultaneously regulate sugar and citric acid contents [22]. However, the cross-regulation between flavor and coloration remains largely unexplored. To date, such a mechanism has only been reported in apple, where *MdMYB1* positively regulates both anthocyanin accumulation and malic acid content [23]. In other horticultural crops, studies are limited, in part due to the scarcity of germplasm that simultaneously accumulates citric acid and anthocyanins.

In this study, we identified a purple wampee (*Clausena lansium*) in the Aurantioideae that simultaneously accumulates high contents of citric acid and anthocyanins in the pulp. Although the purple wampee *Ruby1* has lost its ability to positively regulate anthocyanin biosynthesis, it harbors a PH4-Like (R2R3-MYB) transcription factor that can coordinately positively regulate both citric acid and anthocyanin accumulation. Further analyses revealed that this gene is highly expressed exclusively in purple wampee, while exhibiting extremely low expression in other genera of Aurantioideae. Moreover, this gene exists in the form of R3-MYB due to a shift in its transcription start site in some members of the *Citrus* genus. Promoter activity assays demonstrated that its difference is the key determinant driving this differential expression. This study elucidates the mechanism underlying the concurrent accumulation of citric acid and anthocyanins in purple wampee and further provides deeper insights into the evolutionary trajectories of acidity and pigmentation during *Citrus* diversification. Compared with previous strategies that required the simultaneous manipulation of *PH4* and *Ruby1* to improve fruit acidity and coloration, the discovery of *PH4-Like* offers a more efficient way for improvement of combining these traits.

## Results

### Purple wampee accumulates high contents of citric acid and anthocyanin

**Figure 1:**
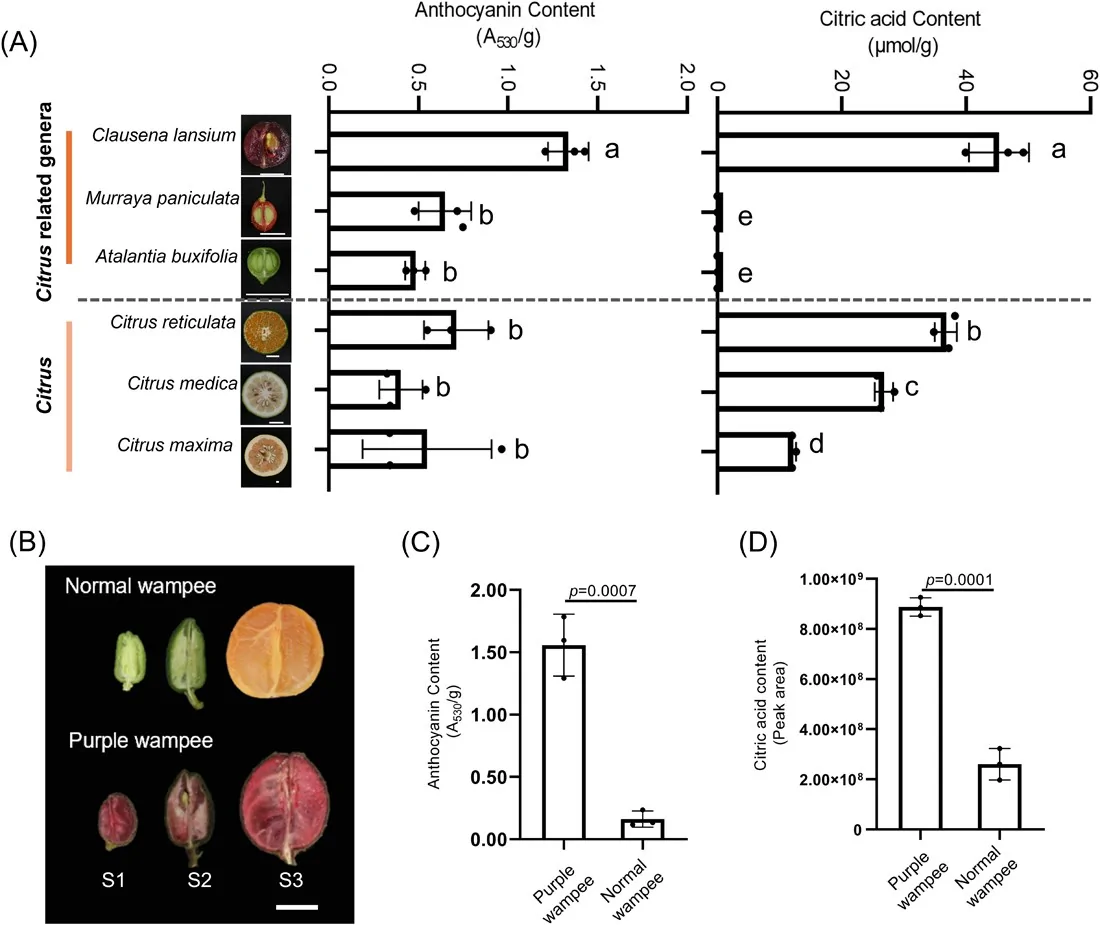
. The purple wampee (*Clausena lansium*) accumulates high contents of both citric acid and anthocyanin. (A) Accumulation patterns of citric acid and anthocyanins in Aurantioideae. The left panel shows the fruit morphology of *Citrus*-related genera and *Citrus*; the middle panel and right panels display the corresponding anthocyanin and citric acid content in pulp, respectively. Purple wampee pulp accumulates high contents of both anthocyanin and citric acid; pulps of other *Citrus-*related genera and the three basic *Citrus* species, *C. reticulata* (Huanglingmiao mandarin), *C. medica* (Qingzang citron), and *C. maxima* (purple pummelo), do not accumulate anthocyanins. Lowercase letters represent significant differences in different fruit pulps by one-way ANOVA followed by the LSD post hoc test (*P* < 0.05). Data are presented as the mean ± standard deviation based on three biological replicates. (B) Early fruit set stage (S1), enlargement stage (S2), and mature stage (S3) of purple wampee and normal wampee fruits. (C and D) Citric acid and anthocyanin content in mature fruit pulp in purple wampee and normal wampee. The data are mean ± standard deviation from three biological replicates. *P* values were determined using a two-tailed Student’s *t*-test. Scale bar = 1 cm.

It is recognized that *Citrus*-related genera lack citric acid and have a bland flavor, with citric acid accumulation emerging only after the divergence of *Citrus* [13]. In this study we identified an early-diverging citrus purple wampee (*Clausena lansium*) from a *Citrus*-related genus that accumulates both high citric acid and anthocyanins in the pulp compared with other *Citrus* genera (Fig. 1A and B). In wampee (*Clausena lansium*) there are cultivated cultivars (normal wampee) with yellow pulp lacking anthocyanin accumulation and showing relatively low citric acid content. In contrast, purple wampee fruit exhibits continuous anthocyanin accumulation after fruit set (Fig. 1B). In addition, blood orange is a well-known cultivar that accumulates anthocyanins in the fruit, but its citric acid content is significantly lower than that of purple wampee (Supplementary Data Fig. S1). These results indicate that purple wampee is a citrus with high levels of both citric acid and anthocyanin.

### Upregulated acid and anthocyanin metabolism-related genes in purple wampee

**Figure 2:**
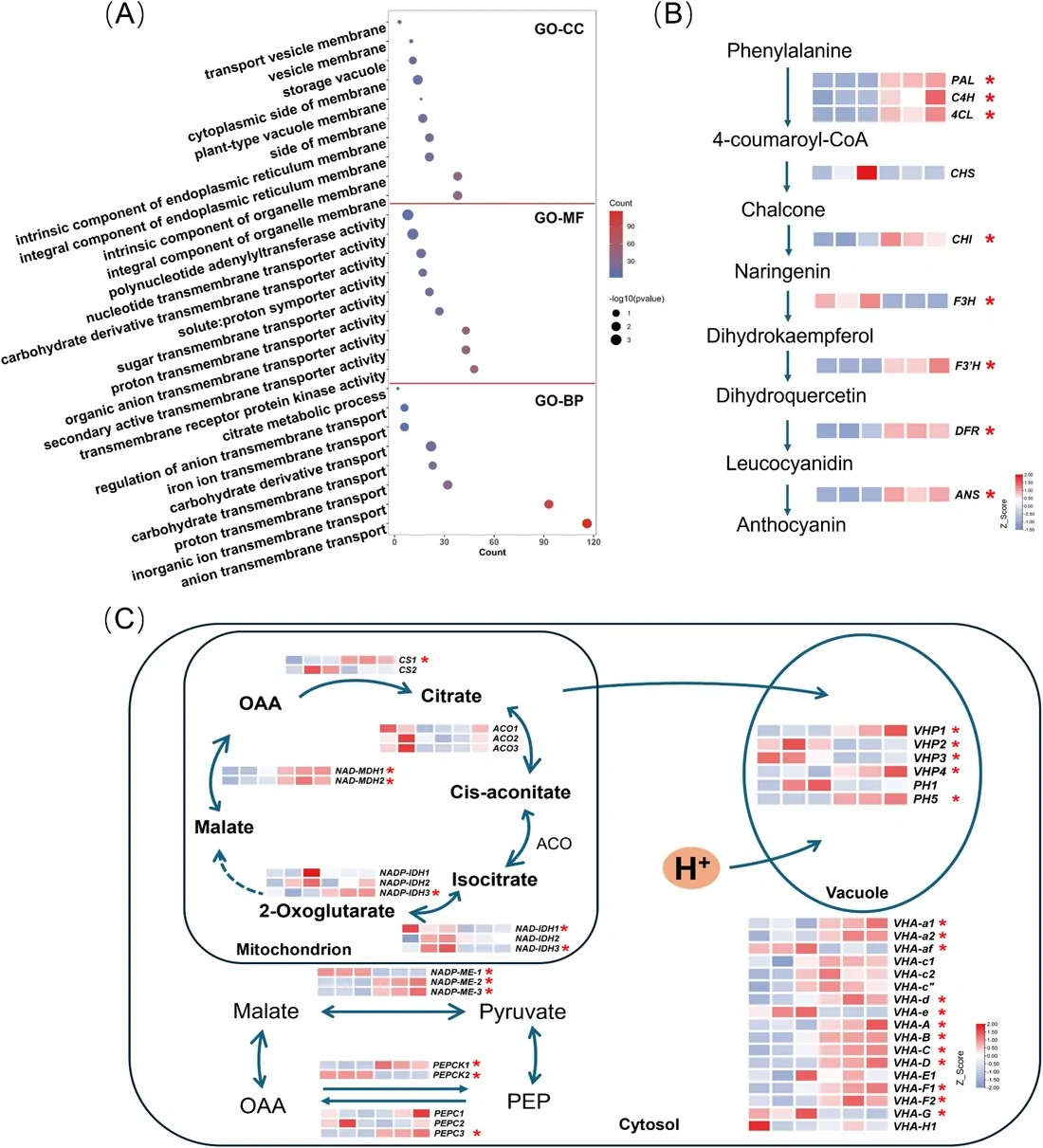
. Enhanced transcription levels in citric acid and anthocyanin metabolism of purple wampee. (A) Functional enrichment analysis of differentially expressed genes in the pulp of purple and normal wampee. (B) Expression profiles of anthocyanin biosynthetic pathway genes in mature pulp (S3) of two types of wampee. (C) Citric acid biosynthesis and transport gene expression in mature pulp (S3) of two types of wampee. The first three columns represent three biological replicates of normal wampee, and the last three represent three biological replicates of purple wampee. Red asterisks indicate significant differences determined by two-tailed *t*-test (*P* < 0.05).

To elucidate the mechanism underlying the significantly higher accumulation of citric acid and anthocyanins in purple wampee, we performed a comparative RNA-seq analysis of mature pulp from purple and normal wampee, revealing that most differentially expressed genes (fold change >1.5 and *P* value <0.05) were related to citric acid metabolism and transmembrane transport (Fig. 2A), suggesting enhanced activity of metabolite transport pathways in purple wampee. To further investigate the expression differences of anthocyanin and citric acid metabolic pathway genes between the two types of wampee fruit, we identified the corresponding pathway genes in the wampee genome (http://citrus.hzau.edu.cn/) and analysed their expression patterns using transcriptome data. The results showed that the expression of key pathway genes for anthocyanin biosynthesis, *Anthocyanidin synthase* (*ANS*), *Dihydroflavonol 4-reductase* (*DFR*), and *Flavonoid 3′-hydroxylase* (*F3′H*) was significantly higher in purple wampee than in normal wampee (Fig. 2B). In addition, most proton pump genes associated with citric acid transport, such as *PH5* and *VHA-a2*, were also significantly upregulated in purple wampee (Fig. 2C). These findings suggest that purple wampee may possess transcription factors that collectively enhance the transcription of both metabolic pathways, thereby leading to the high accumulation of citric acid and anthocyanins.

### Identification of transcription factors promoting the coordinated accumulation of citric acid and anthocyanins in purple wampee

**Figure 3:**
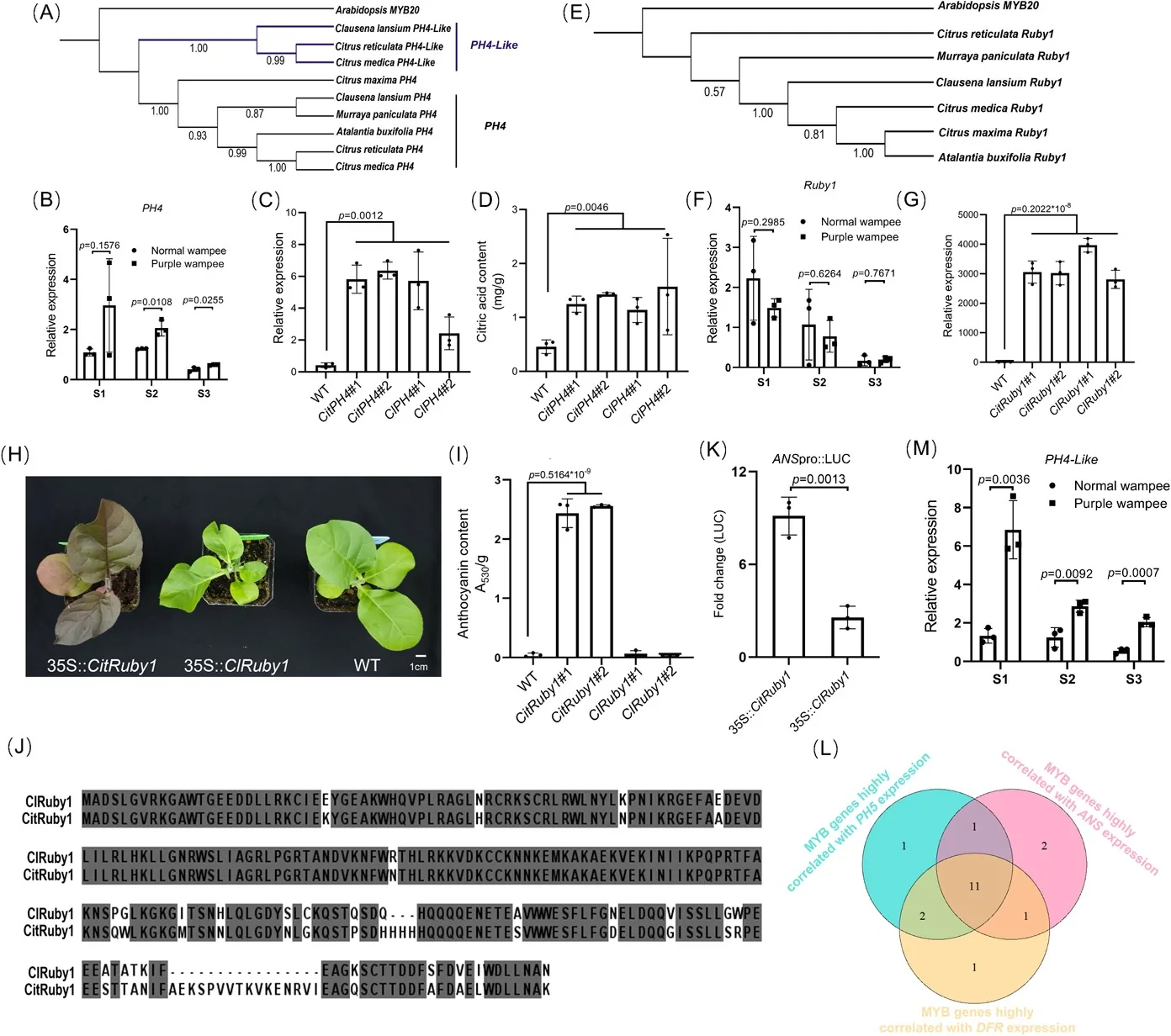
. Screening of transcription factors promoting the coordinated accumulation of citric acid and anthocyanins. (A) Phylogenetic analysis of *PH4* in Aurantioideae. The phylogenetic tree was constructed using the neighbor-joining (NJ) method using MEGA-x. (B) Expression of *PH4* in pulp of purple and normal wampee at three developmental stages. (C and D) Stable overexpression of citrus *PH4* (*CitPH4*) and purple wampee *PH4* (*ClPH4*) in citrus, and corresponding citric acid content. (E) Phylogenetic analysis of *Ruby1* in Aurantioideae. The phylogenetic tree was constructed using the NJ method using MEGA-x. (F) Expression of *Ruby1* in pulp of purple and normal wampee at three developmental stages. (G and H) Stable overexpression of citrus *Ruby1* (*CitRuby1*) and purple wampee *Ruby1* (*ClRuby1*) in tobacco (*Nicotiana tabacum*). (I) Stable overexpression of *CitRuby1* and *ClRuby1* in tobacco results in differential anthocyanin accumulation. (J) Comparative analysis of the amino acid sequences of CitRuby1 and ClRuby1. (K) Comparative analysis of the differential activation of *Anthocyanidin synthase* (*ANS*) by CitRuby1 and ClRuby1. (L) Intersection with MYB genes showing strong expression correlation with *PH5*, *Dihydroflavonol 4-reductase* (*DFR*), and *ANS*. (M) Expression of *PH4*-*Like* in pulp of purple and normal wampee at three developmental stages. All data are mean ± standard deviation based on three biological replicates. *P* values were determined using a two-tailed Student’s *t*-test.

To further elucidate the mechanism underlying the simultaneous accumulation of citric acid and anthocyanins in purple wampee, we identified the transcription factor PH4 (Fig. 3A), which positively regulates citric acid accumulation. Its expression was consistently higher in the pulp of purple wampee than in that of normal wampee across three developmental stages (Fig. 3B). Then we generated transgenic citrus showing stable overexpression of wampee *PH4* (*ClPH4*) and *Citrus PH4* (*CitPH4*) (Fig. 3C). Analysis of citric acid content in this transgenic citrus revealed that overexpression of both *ClPH4* and *CitPH4* significantly increased citric acid accumulation (Fig. 3D). In addition, Ruby1 has been reported as a major MYB regulator promoting anthocyanin accumulation (Fig. 3E). However, no significant differences in *Ruby1* expression were observed between purple wampee and normal wampee pulp across the three developmental stages (Fig. 3F). Overexpression of purple wampee *Ruby1* (*ClRuby1*) and citrus *Ruby1* (*CitRuby1*) in tobacco (*Nicotiana tabacum*) (Fig. 3G) revealed that *CitRuby1* positively regulated anthocyanin accumulation, whereas overexpression of *ClRuby1* neither increased anthocyanin content nor resulted in visible pigmentation (Fig. 3H and I). Further analysis showed multiple differences between the amino acid sequences of ClRuby1 and CitRuby1 (Fig. 3J), and ClRuby1 exhibited significantly lower activation of *ANS* compared with CitRuby1 (Fig. 3K and Supplementary Data Fig. S2).

To further identify transcription factors that simultaneously promote anthocyanin and citric acid accumulation, we first identified 256 MYB genes in the wampee genome (Supplementary Data Table S2). Transcriptome analysis subsequently revealed 44 MYB genes that were more highly expressed in purple wampee pulp than in normal wampee pulp at the ripening stage (S3) (Supplementary Data Table S3). Subsequently, expression correlation analyses were performed between these 44 MYB genes and *PH5*, *ANS*, and *DFR*. The top 15 MYB genes showing the strongest correlations with each of these genes were selected, and intersection analysis of these sets yielded 11 candidate MYB genes (Fig. 3L). Finally, expression profiling of these MYBs at the S1 and S2 developmental stages in both wampee types showed that one gene, *HP011110*, was consistently and significantly more highly expressed in purple wampee pulp than normal wampee pulp at both stages (Fig. 3M and Supplementary Data Fig. S3). Sequence analysis further indicated that *HP011110* is a *PH4-Like* gene (Fig. 3A). Therefore, this gene was selected as the primary candidate for further investigation.

### *PH4-Like* positively regulates citric acid and anthocyanin accumulation

**Figure 4:**
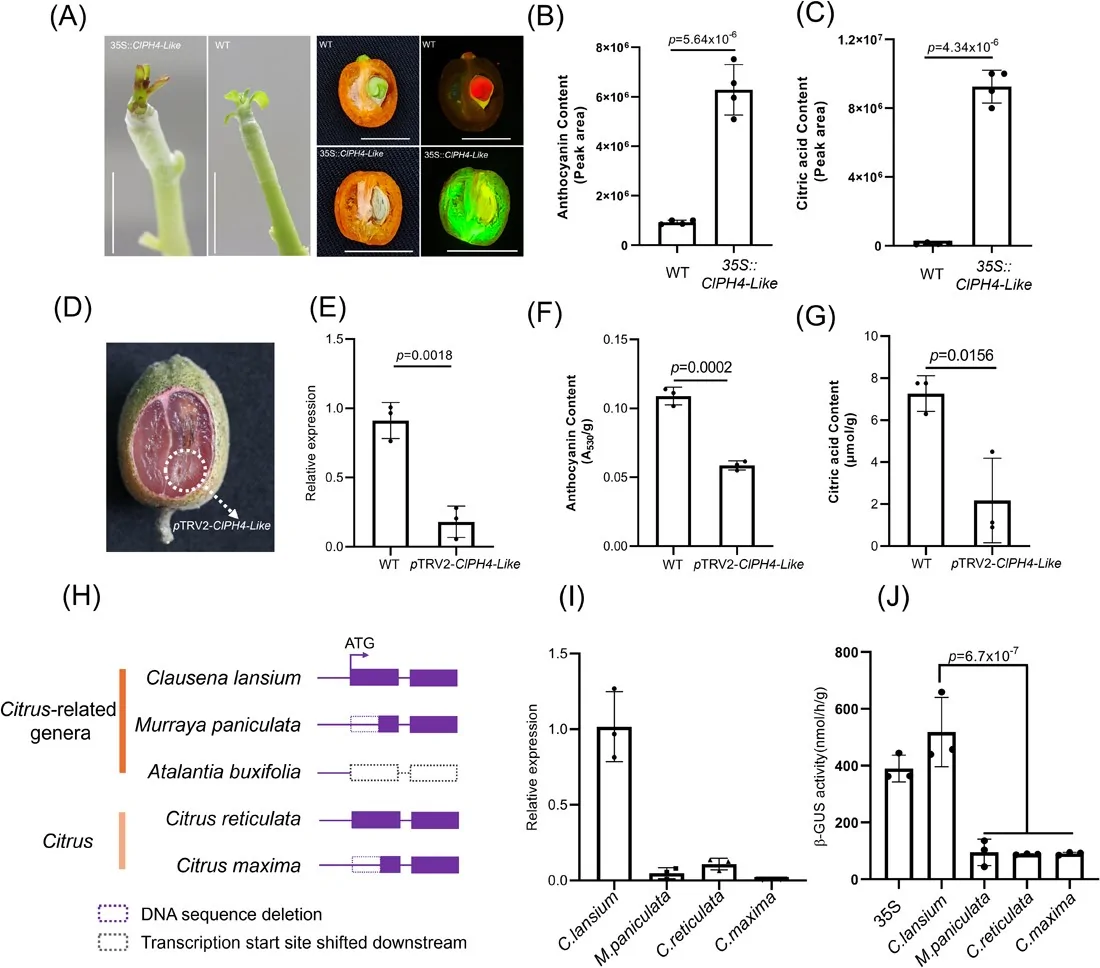
. *PH4-Like* positively regulates the accumulation of both citric acid and anthocyanin. (A) Stable overexpression of *PH4-Like* leads to anthocyanin accumulation in young leaves. (B, C) Measurement of anthocyanin and citric acid content in citrus fruits overexpressing *PH4-Like*. For WT we used four independent samples; the transgenic citrus samples were pooled from fruits of two overexpression lines and divided into two independent samples for extraction. (D) Transient silencing of *PH4-Like* in purple wampee fruit. (E) Expression analysis of *PH4-Like* after transient silencing. Each group consisted of three biological replicates. (F, G) Measurement of anthocyanin and citric acid content in purple wampee fruits with transient silencing of *PH4-Like*. Each group consisted of three biological replicates. (H, I) Gene structure of *PH4-Like* and expression analysis in the fruits of *Citrus*-related genera and *Citrus*. Each group consisted of three biological replicates. (J) Promoter activity analysis of *PH4-Like* in *Citrus*-related genera and *Citrus*. Each group consisted of three biological replicates. All data are mean ± standard deviation. *P* values were determined using a two-tailed Student’s *t*-test. Scale bar = 1 cm.

To investigate whether *PH4-Like* affects the accumulation of citric acid and anthocyanins, the purple wampee *PH4-Like* (*ClPH4-Like*) was stably overexpressed in citrus (Fig. 4A). The results showed that overexpression of *ClPH4-Like* significantly increased the contents of anthocyanins and citric acid (Fig. 4B and C), accompanied by noticeable pigmentation (Fig. 4A). To further elucidate its function, *ClPH4-Like* was successfully transiently silenced in purple wampee pulp, resulting in an ~80.28% reduction in expression (Fig. 4E), visibly lighter pigmentation (Fig. 4D), and significant decreases in anthocyanin and citric acid contents (Fig. 4F and G). These results indicate that *ClPH4-Like* positively regulates the accumulation of citric acid and anthocyanins in purple wampee.

To further investigate the sequence and expression characteristics of *PH4-Like* in *Citrus*-related genera and *Citrus*, we performed a detailed analysis. The results showed that, apart from purple wampee, *PH4-Like* is completely lost in the *Atalantia buxifolia* chromosome, while in *Murraya paniculata* and *Citrus maxima* a downstream shift of the transcription start site leads to the formation of an R3-MYB-type (*PH4-Like^Short^*) (Fig. 4H). Moreover, *PH4-Like* expression was significantly higher in the fruit pulp of purple wampee than in that of other citrus (Fig. 4I). Promoter activity assays further demonstrated that the *ClPH4-Like* promoter was significantly more active than those from other citrus (Fig. 4J). Additionally, stable overexpression of the *PH4-Like^Short^* in citrus severely impaired plant growth and development (Supplementary Data Fig. S4). Collectively, these results indicate that *ClPH4-Like* uniquely promotes the simultaneous accumulation of citric acid and anthocyanins in purple wampee.

### *PH4-Like* positively regulates the transcription of acid and anthocyanin pathway genes

**Figure 5:**
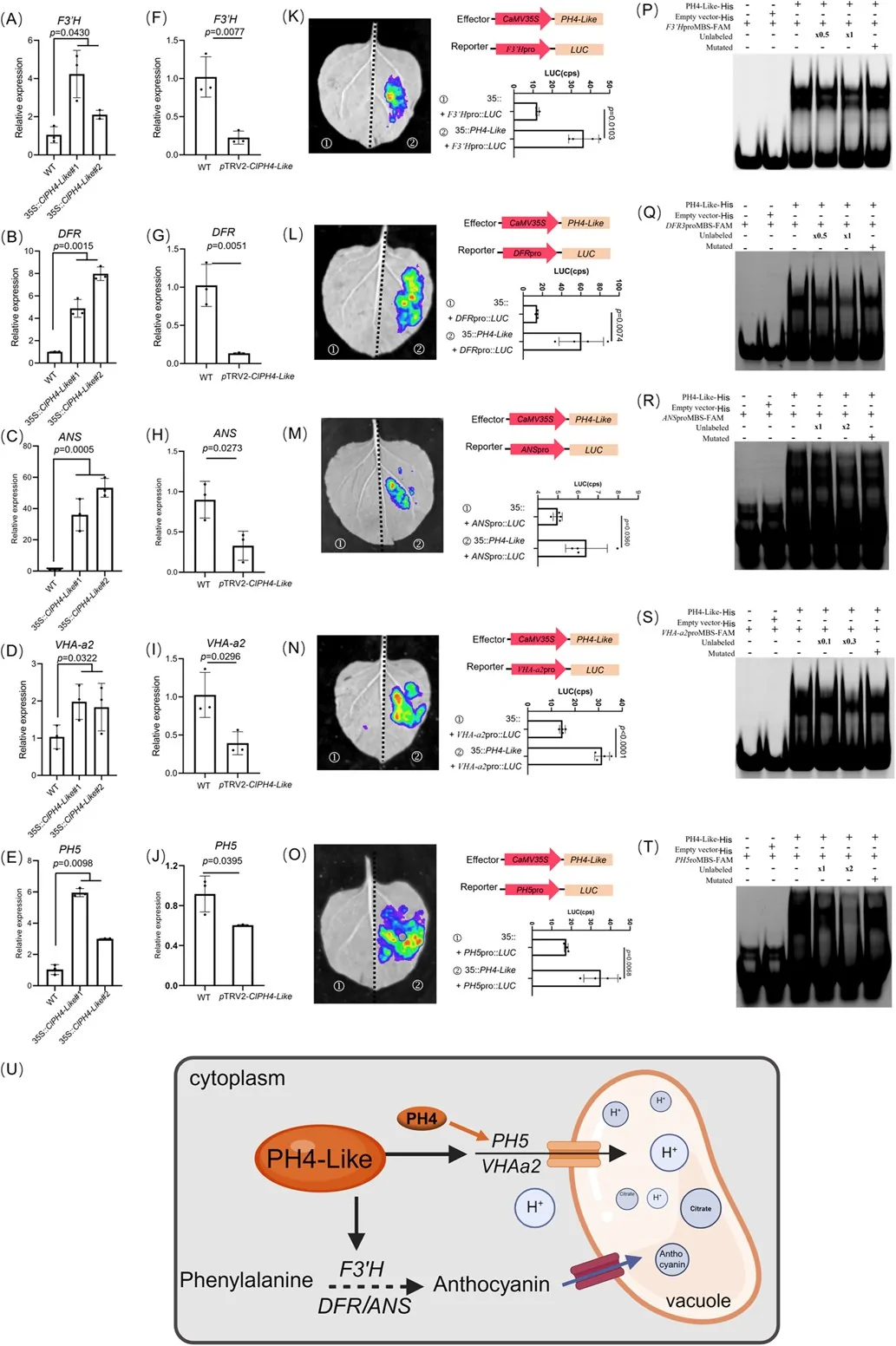
. *PH4-Like* positively regulates the expression of citric acid and anthocyanin-related pathways genes. (A–J) Expression changes of *F3′H*, *DFR*, *ANS*, *VHA-a2*, and *PH5* in citrus fruit with stable overexpression of *PH4-Like* and in purple wampee pulp with *PH4-Like* silencing. (K–O) Luciferase reporter assays confirmed that PH4-Like can activate the transcription of *F3′H*, *DFR*, *ANS*, *VHA-a2*, and *PH5*. On the left in each panel is shown in vivo imaging in tobacco leaves to detect the activating effects. The top right panel shows a diagram of the effector and reporter constructs. The bottom right panel shows quantitative fluorescence intensity. Four independent tobacco leaves were used as replicates. (P–T) PH4-Like binds the *F3′H*, *DFR*, *ANS*, *VHA-a2* and *PH5* promoter. Unlabeled probe refers to a probe that is not labeled with FAM. Mutated probe refers to a probe in which all the binding sites labeled with FAM have been replaced with adenine. (U) Proposed model of *PH4-Like* gene coordinately promoting citric acid and anthocyanin accumulation in purple wampee. In the purple wampee pulp, *PH4-Like* is highly expressed and promotes the expression of proton pump-related genes (*VHA-a2* and *PH5*) and anthocyanin biosynthetic genes (*F3′H*, *DFR*, and *ANS*). Orange arrows indicate previously reported regulatory relationships. Data are mean ± standard deviation. *P* values were determined using a two-tailed Student’s *t*-test.

To investigate the molecular mechanism by which *PH4-Like* regulates citric acid and anthocyanin accumulation, we first analysed the transcriptional abundance of key pathway genes. The results showed that the expression of *Flavonoid 3′-hydroxylase* (*F3′H*), *Dihydroflavonol 4-reductase* (*DFR*), *Anthocyanidin synthase* (*ANS*), and the proton pump genes *PH5* and *VHA-a2* were significantly upregulated in *PH4-Like*-overexpressing citrus pulp (Fig. 5A–E). Moreover, these expression levels were also significantly downregulated in the pulp of *PH4-Like*-silenced purple wampee (Fig. 5F–J).

Furthermore, luciferase (LUC) assays revealed that PH4-Like significantly activated the transcription of these genes (Fig. 5K–O). EMSA experiments demonstrated that PH4-Like bound to the promoters of *F3′H*, *DFR*, *ANS*, *VHA-a2*, and *PH5* (Fig. 5P–T). These results indicate that PH4-Like positively regulated the expression of acid-related proton pump genes and anthocyanin biosynthetic genes, thereby coordinately promoting the accumulation of citric acid and anthocyanins in purple wampee pulp (Fig. 5U).

## Discussion

This study characterized a novel purple wampee in Aurantioideae that simultaneously accumulates high levels of citric acid and anthocyanin in the fruit pulp. We uncovered a new transcription factor, PH4-Like, which positively regulates the accumulation of both citric acid and anthocyanins in purple wampee by regulating the transcription abundance of *F3′H*, *DFR*, *ANS*, *VHA-a2*, and *PH5*. By analysing the sequence and expression profiles of this gene across different citrus species, the results demonstrated that the higher promoter activity of *PH4-Like* in purple wampee underlies its uniquely elevated expression in the purple wampee fruit pulp.

During citrus diversification, the accumulation of citric acids and anthocyanin has progressively tended toward regulation by specialized transcription factors. The S6 subfamily of the MYB family is broadly conserved across the plant for promoting anthocyanin accumulation [24–27]. In citrus, *Ruby1* has undergone clear functional differentiation. In the ancestral citrus species *Atalantia buxifolia*, *Ruby2*–*Ruby1* forms a gene cluster that regulates anthocyanin accumulation in different tissues [2]. After divergence in *Citrus*, *Ruby2* lost its function in promoting anthocyanin biosynthesis, leaving *Ruby1* solely responsible for anthocyanin accumulation across tissues [28]. This study further investigated the evolutionary patterns of anthocyanins in Aurantioideae and their underlying mechanisms. In purple wampee, which diverged earlier than *Atalantia buxifolia* [13], *Ruby2* is absent, and *Ruby1* lost its capacity to positively regulate anthocyanin accumulation, with *PH4-Like* instead taking on this role. As diversification proceeded, *PH4-Like* was lost in *Atalantia buxifolia* but reappeared in the *Citrus* genus, although it no longer retained its function, while *Ruby1* gradually evolved to become exclusively responsible for anthocyanin accumulation. In purple wampee, citric acid accumulation is co-regulated by *PH4* and *PH4-Like*, which may account for its relatively high citric acid content. Following further diversification within Aurantioideae, the loss of *PH4-Like* function led to citric acid accumulation being exclusively regulated by *PH4*.

A deeper understanding of pleiotropy and the discovery of genes that coordinately regulate key traits are essential for optimizing breeding parent selection and identifying targets for trait improvement. For example, the rice *Ideal Plant Architecture* (*IPA1*) gene has been extensively studied not only for its role in regulating plant architecture [29], but also for its mechanisms in enhancing yield and disease resistance [30, 31], thereby laying an important foundation for the coordinated improvement of rice architecture, yield, and resistance through *IPA1*. In addition, the key gene *WOS1*, which links seed oil content and size in soybean, has also been identified, providing a valuable genetic target for soybean improvement [32]. While such research has made substantial progress in food crops and contributed to breeding, it remains underexplored in horticultural crops, particularly perennial fruit trees, the long juvenile phase of which restricts further research on the pleiotropy of key genes. The discovery of *PH4-Like* is of great significance for improving both the color and taste of citrus fruit. *PH4* and *PH4-Like* share a relationship like that of *MYB5a* and *MYB5b* in grapevine [33–35], as they are all paralogous genes. However, in grapevine it remains unclear whether *MYB5a* and *MYB5b* affect tartaric acid accumulation. Likewise, although the strawberry gene *FaMYB5* has been reported to positively regulate the phenylpropanoid pathway and citric acid accumulation separately [4, 5], it remains unclear whether such functional divergence and alternation exist within the Rosaceae family. Therefore, in most horticultural crops improving both taste and pigmentation still requires the distinct regulators that regulate each trait independently. This undoubtedly increases the complexity of breeding. The discovery of *PH4-Like* provides a simplified strategy for improving citrus coloration and acidity, enabling the development of new citrus cultivars with appealing color and suitable acidity through the modification of a single gene using approaches such as gene editing or somatic hybridization.

## Materials and methods

### Plant materials and metabolite determination

Plant materials used in this study were collected from the germplasm orchard of the National Citrus Breeding Center. Purple wampee fruit were sampled from an orchard in Daojia Village, Xiangqi Town, Teng County, Wuzhou City, Guangxi, China. The citric acid content of different Aurantioideae resources was determined using a Solarbio citric acid assay kit (BC2155). Anthocyanin content was primarily assessed by measuring absorbance at 530 nm, following previously described methods [36]. Citric acid content in citrus with overexpression of PH4 was measured by GC, following previously reported methods [37].

### RNA extraction and RT–PCR analysis

Total RNA was extracted from plant tissues using the Trizol Iso Plus reagent (Takara) according to the manufacturer’s instructions. cDNA was synthesized from RNA using the HiScript II Reverse Transcriptase Kit (Vazyme Biotech). Quantitative real-time PCR (qRT–PCR) was performed on a LightCycler 480 system (Roche) with Hieff qPCR SYBR Green Master Mix (YEASEN). Relative gene expression was analysed using the 2^−ΔΔCt^ method. Primer sequences are listed in Supplementary Data Table S1.

### Sequence analysis, vector construction, and plant transformation

The sequences of the *PH4*, *PH4-Like*, and *Ruby1* genes in the Aurantioideae were obtained from the Citrus Genome Database (http://citrus.hzau.edu.cn/). A phylogenetic tree was constructed using the neighbor-joining (NJ) method with MEGA-x. Genome-wide identification of the MYB gene family in the wampee genome was based on a hidden Markov model.

After cloning the CDS of *Citrus PH4*, *Clausena lansium PH4*, *Citrus Ruby1*, *Clausena lansium Ruby1*, and *Clausena lansium PH4*-*Like*, they were linked to the 35S-driven overexpression vector 1300 by homologous recombination. The CDS of *Clausena lansium PH4*-*Like* was subsequently linked to the virus-induced gene silencing (VIGS) vector *p*TRV2 through a homologous recombination reaction. The constructed vectors were introduced into *Agrobacterium* strain EHA105. Stable transformation was performed via epicotyl infiltration in Carrizo citrange (*Citrus sinensis* ‘Washington’ sweet orange × *Poncirus trifoliata*) [38] and leaf disk infiltration in tobacco (*Nicotiana tabacum*) [39]. Transient transformation of purple wampee pulp was carried out following previously described protocols [40]. *Agrobacterium* (EHA105) carrying pTRV2-*PH4-Like* was resuspended in a solution containing MgCl₂ (2 mg/L) and MES (2 mg/L), and the OD_600_ was adjusted to 0.6. Approximately 300 μl of the suspension was injected into purple wampee pulp using a 1-ml syringe. The fruits were then incubated in the dark at 28°C for 5 days, after which samples were collected to assess *PH4-Like* expression and measure citric acid and anthocyanin contents.

### Dual-luciferase reporter assay

The promoters of *F3′H*, *DFR*, *ANS*, *VHA-a2*, and *PH5* were amplified in purple wampee and then ligated to the LUC reporter vector by homologous recombination to drive LUC expression. The *PH4-Like* gene driven by the 35S promoter was used as the effector. These plasmids were individually transformed into *Agrobacterium* strain GV3101. The effector and reporter strains were mixed at a volume ratio of 5:1 and infiltrated into leaves of tobacco leaves (*Nicotiana benthamiana*). After 3 days, bioluminescence signals were detected using a NightShade imager (LB985) and quantified. The primers used are listed in Supplementary Data Table S1.

### Electrophoretic mobility shift assay

The full-length CDS of *PH4-Like* was cloned into the pET-32a(+) vector to generate a fusion construct expressing 6 × His-*CitPH4-Like*-6 × His. The recombinant plasmid was introduced into *Escherichia coli* BL21 (DE3) for protein expression, which was performed as previously described [41]. MYB binding sites within the promoters of *F3′H*, *DFR*, *ANS*, *VHA-a2*, and *PH5* were predicted using the JASPAR database (https://jaspar.elixir.no/). FAM-labeled DNA probes were subsequently synthesized by Sangon Biotech (Shanghai, China). Electrophoretic mobility shift assays (EMSAs) were performed with a commercial kit (Beyotime), following the manufacturer’s instructions. Images were captured using a multifunctional imaging system. Primer sequences are provided in Supplementary Data Table S1.

## Supplementary Material

Web_Material_uhag182

## Data Availability

The sequence of purple wampee *PH4-Like* has been submitted to GenBank under accession number PV927266.
