# Supplementary material for: ClPH4-like coordinately regulates citric acid and anthocyanin accumulation in purple wampee
Source: Hortic Res. 2026 May 4;13(10):uhag182. doi: 10.1093/hr/uhag182 (PMC13610575; doi:10.1093/hr/uhag182)
Supplement: Supplementary file 1 [file web_material_uhag182.zip › Supplementary Figures .docx]

Supporting information

Figure S1. Morphology of purple wampee and blood orange, and measurement of their citric acid content. (A) growth status of purple wampee in the cultivation orchard. (B) Top and bottom, citric acid content in the fruits of purple wampee and blood orange, respectively

Figure S2. Comparison of the differential activation of *Anthocyanidin synthase* (*ANS*) by CitRuby1 and ClRuby1. Left panel, schematic of the injection layout. Right panel, resulting activation effects.

Figure S3. Expression patterns of candidate differentially expressed MYB genes in the pulp of purple and normal wampee at two developmental stages. Data are presented as the mean ± SD, *p* values were determined using a two-tailed Student’s t-test.

Figure S4. Overexpression of *PH4-Like^Short^* severely affects citrus growth and development. Right panel, morphology of *PH4-Like^Short^* -overexpressing citrus plants at two years.
